# Pediatric Stroke: A Review

**DOI:** 10.1155/2011/734506

**Published:** 2011-12-27

**Authors:** Daniel S. Tsze, Jonathan H. Valente

**Affiliations:** ^1^Department of Pediatrics, Division of Pediatric Emergency Medicine, College of Physicians and Surgeons, Columbia University, New York, NY 10032, USA; ^2^Department of Emergency Medicine and Pediatrics, Warren Alpert Medical School of Brown University, Providence, RI 02903, USA

## Abstract

Stroke is relatively rare in children, but can lead to significant morbidity and mortality. Understanding that children with strokes present differently than adults and often present with unique risk factors will optimize outcomes in children. Despite an increased incidence of pediatric stroke, there is often a delay in diagnosis, and cases may still remain under- or misdiagnosed. Clinical presentation will vary based on the child's age, and children will have risk factors for stroke that are less common than in adults. Management strategies in children are extrapolated primarily from adult studies, but with different considerations regarding short-term anticoagulation and guarded recommendations regarding thrombolytics. Although most recommendations for management are extrapolated from adult populations, they still remain useful, in conjunction with pediatric-specific considerations.

## 1. Background

Stroke is a neurological injury caused by the occlusion or rupture of cerebral blood vessels. Stroke can be ischemic, hemorrhagic, or both. Ischemic stroke is more frequently caused by arterial occlusion, but it may also be caused by venous occlusion of cerebral veins or sinuses. Hemorrhagic stroke is the result of bleeding from a ruptured cerebral artery or from bleeding into the site of an acute ischemic stroke (AIS). 

AIS accounts for about half of all strokes in children, in contrast to adults in whom 80–85% of all strokes are ischemic [1, 2]. Children also have a more diverse and larger number of risk factors for stroke that differ significantly from adults which are predominated by hypertension, diabetes, and atherosclerosis [3, 4].

Pediatric stroke leads to significant morbidity and mortality. Roughly 10–25% of children with a stroke will die, up to 25% of children will have a recurrence, and up to 66% will have persistent neurological deficits or develop subsequent seizure disorders, learning, or developmental problems [3, 5, 6]. Given the onset of impairment during childhood and the effect on quality of life for the child and family, the economic and emotional costs to society are amplified.

Early recognition of pediatric stroke should lead to more rapid neurological consultation, imaging, treatment, and improved outcomes. In this article, we will review the epidemiology, clinical presentation, differential diagnosis, risk factors and causes, and management of pediatric stroke. Neonatal stroke will not be discussed in this paper.

## 2. Epidemiology

A stroke or cerebral vascular accident (CVA) in children is typically considered to be a rare event. The reported incidence of combined ischemic and hemorrhagic pediatric stroke ranges from 1.2 to 13 cases per 100,000 children under 18 years of age [1, 7–15]. However, pediatric stroke is likely more common than we may realize since it is thought to be frequently undiagnosed or misdiagnosed. This may be due to a variety of factors including a low level of suspicion by the clinician and patients who present with subtle symptoms that mimic other diseases. This, in turn, can lead to a delay in the diagnosis of stroke. In one report, 19 out of 45 children with a stroke did not receive a correct diagnosis until 15 hours to 3 months after initial presentation [16]. Another study demonstrated up to a 28-hour delay in seeking medical attention from the onset of symptoms and a 7.2-hour average delay after presentation before any brain imaging was done [17]. However, the reported incidence of pediatric stroke has more than doubled from prior decade estimates [18]. This may be due to a combination of increased survival in children with risk factors for stroke, such as congenital heart disease, sickle cell disease, and leukemia, and increased awareness [4, 6, 18].

Stroke is more common in boys than girls, even after controlling for differences in frequency of causes such as trauma. There appears to be a predominance of stroke in black children [9]. This difference remains true even after accounting for sickle cell disease patients with stroke [15].

## 3. Clinical Presentation

There are some generalizations that can be made as to how strokes present in children (Table 1). AIS most often presents as a focal neurologic deficit. Hemiplegia is the most common focal manifestation, occurring in up to 94% of cases [1, 10, 19–21]. Hemorrhagic strokes most commonly present as headaches or altered level of consciousness, and are more likely to cause vomiting than in AIS [1, 10, 22]. Seizures are common in both ischemic and hemorrhagic strokes. They occur in up to 50% of children with strokes, are not restricted to any age group, and are not limited to any specific seizure type [23].

There can be significant differences in the clinical presentation based on the child's age. The younger the child, the more nonspecific their symptoms may be. Perinatal strokes are more likely to initially present with focal seizures or lethargy in the first few days after birth [18, 24]. Although focal neurological deficits from these events may not develop until weeks or months later, infants within the first year of life can still present acutely with lethargy, apnea spells, or hypotonia [5, 17, 25]. Toddlers can also present with protean symptoms such as deterioration of their general condition, increased crying and sleepiness, irritability, feeding difficulty, vomiting, and sepsis-like symptoms with cold extremities [26]. Older children demonstrate more specific neurological defects similar to adults. These include hemiparesis, language (e.g., aphasia) and speech difficulties, visual deficits, and headache [10, 19, 20, 27–29]. If symptoms last less than 24 hours, they are defined as a transient ischemic attack (TIA) [30]. Deficits are frequently brief and may resolve as quickly as within one hour [18]. Older children may even be able to report prior episodes of suspicious signs or symptoms. Recent data suggests that 33% of children with arterial strokes had preceding TIAs that were undiagnosed at that time [18].

Specific types of stroke will also present differently in each age group. For example, venous sinus thrombosis can present in all ages with fever and lethargy, but young infants can present with a history of decreased oral intake or respiratory distress [31–34]. Physical examination may reveal dilated scalp veins, eyelid swelling, or a large anterior fontanelle whereas an older child would likely present with more slowly progressing signs, such as vomiting, headache, or any other signs of increased intracranial pressure [31–34]. A subarachnoid hemorrhage can also present as irritability and a bulging fontanelle in infants, but should be suspected in older children complaining of sudden acute onset headache, neck pain, meningismus, or photophobia [35].

The clinical presentation is also useful for localizing the lesion. The majority of pediatric ischemic strokes occur in the distribution of the middle cerebral artery, which results in hemiplegia with upper limb predominance, hemianopsia, or dysphasia. Primarily lower extremity weakness would suggest anterior cerebral artery involvement whereas vertigo, ataxia, and nystagmus are consistent with an ischemic event in the posterior circulation [19–21, 36]. Bulbar dysfunction and dysarthria points towards lower brainstem involvement whereas aphasia suggests involvement of the basal ganglia, thalamus, or cerebral hemispheres. If the hemispheres are involved, then the eyes will look towards the lesion, rather than away as if the brainstem were involved.

## 4. Differential Diagnosis

There are many other diseases that may mimic a stroke. Complicated migraines can cause focal neurologic symptoms that typically resolve within 24 hours, and should be considered if there is a family history of migraine or hemiplegic migraine [10]. Focal seizures can result in subsequent transient postictal hemiparesis (Todd's Paresis), but stroke should be considered if the duration of the deficit is prolonged relative to the duration of the preceding seizure. Intracranial neoplasms should be considered, as well as intracranial infections such as meningitis, brain abscess, and herpes simplex encephalitis [4, 37]. Although rare, alternating hemiplegia is a possibility, especially if there is a distinct history of episodes of hemiplegia that last rarely longer than a day, alternate between sides, and present in a child with progressive developmental regression [23]. Common metabolic abnormalities like hypoglycemia can cause focal, stroke-like deficits [38]. Uncommon metabolic disorders such as MELAS (mitochondrial myopathy, encephalopathy, lactic acidosis, and stroke), which is inherited, can also cause stroke-like symptoms, without an actual ischemic or hemorrhagic event [2, 39].

## 5. Risk Factors and Causes

The majority of signs and symptoms of stroke are nonspecific, and can be easily attributed to other causes. One way to avoid delays or misdiagnoses would be to identify risk factors for stroke that would prompt more aggressive and timely investigation. Multiple risk factors are often present in as many as 25% of children with stroke, which means further investigations are warranted even when one risk factor has been identified [18, 24].

### 5.1. Cardiac

Cardiac disease is the most common cause of stroke in childhood, accounting for up to a third of all AIS [4]. In children with a cardiac repair or catheterization, nearly 50% of strokes occur within 72 hours. Long-standing cyanotic lesions cause polycythemia and anemia, which both increase the risk of thromboembolism and cerebral infarction [2]. Embolic clots can arise in children with cardiomyopathies, rheumatic heart disease, prosthetic valves, or valvular vegetation from endocarditis [2, 24]. A patent foramen ovale (PFO) can occur in as many as 35% of people between ages 1 and 29 years, and may serve as a portal for venous embolic events to pass from the right to left side of the heart [40]. 

### 5.2. Hematologic

Sickle cell disease (SCD) is a very common cause of pediatric stroke, occurring in 285 cases per 100,000 affected children [1]. Strokes may occur as early as 18 months of age, but most children present after five years of age [41]. AIS is more common in the younger age group whereas hemorrhagic strokes occurs more frequently in older children and adults [42]. Strokes may occur in the absence of pain or aplastic crises [43]. Two-thirds of children with SCD who have had previous strokes but remain untreated will have a recurrence [44]. The exact pathophysiology is not entirely clear, although it likely involves elements of anemia, microvascular occlusion, stasis causing reperfusion injury physiology, and endothelial dysfunction [45].

Prothrombotic disorders have been identified in 30 to 76% of patients experiencing arterial or venous events, and should be suspected if there is a family history of early onset AIS (particularly if under 55 years old), heart disease, pulmonary embolism, or deep vein thrombosis events [24, 46–49]. Acquired prothrombotic disorders secondary to deficiencies in proteins C and S may occur in children with renal and liver disease, including nephrotic syndrome with loss of coagulation factors [2, 50]. Protein C deficiency has also been reported in children taking valproate [51]. Hemorrhagic strokes can arise from both Factor VII and factor VIII deficiency [52, 53]. Iron deficiency anemia has been reported in children with both AIS and venous thrombosis with no other apparent etiology [24, 54, 55].

### 5.3. Infection

Varicella infection within the past year can result in basal ganglia infarction [56, 57]. HIV infection can cause stroke secondary to HIV-induced vasculitis, vasculopathy with subsequent aneurysms, or hemorrhage in the context of immune thrombocytopenia [58, 59]. More commonly associated organisms include mycoplasma and chlamydia, as well as enterovirus, parvovirus 19, influenza A, coxsackie, Rocky Mountain spotted fever, or cat scratch disease [58, 60]. Five to twelve percent of children with bacterial meningitis, TB meningitis, and viral encephalitis will have a stroke due to local vasculitis and thrombosis. A history of drinking raw milk or visiting a farm may point to a diagnosis of neurobrucellosis [61]. Head and neck infections, such as mastoiditis or periorbital infections, remain important causes of CVT [2, 31]. 

### 5.4. Vascular

Arteriovenous malformations (AVM) are the most common cause of hemorrhagic stroke after infancy, but can also cause thrombotic stroke [8, 10, 62]. AVM may be associated with neurocutaneous syndromes such as Osler-Weber-Rendu syndrome (i.e., hereditary hemorrhagic telangiectasia), Sturge-Weber disease, neurofibromatosis, or von Hippel-Lindau syndrome. Moyamoya is another important vascular cause of childhood stroke and is associated with conditions such as Down syndrome, neurofibromatosis, and sickle cell disease [24, 30]. 

### 5.5. Syndromic and Metabolic Disorders

Although rare, children with Marfan syndrome are at risk of ischemic neurovascular complications [63]. Children with tuberous sclerosis have a higher risk of embolic events, and may also have hemorrhagic strokes secondary to hypertension, hemorrhage into a tumor, or rupture of an abnormal vessel [39]. Homocysteinuria can cause AIS and should be suspected in the presence of mental retardation associated with lens dislocation and occasionally pectus excavatum [64]. Nutritional deficiencies of folic acid or vitamin B12 may also cause hyperhomocysteinemia, leading to stroke [2]. There is an elevated risk for AIS secondary to thrombosis and premature arteriosclerosis [65], the latter of which is also caused by familial lipoprotein disorders [66–69].

### 5.6. Vasculitis

Cerebral vasculitis is a less common cause of stroke in children, and is more common in children older than 14 years of age [8]. Although idiopathic vasculitis is most often diagnosed, signs and symptoms of systemic vasculitides with Kawasaki disease, Henoch-Sch$\ddot{\text{o}}$nlein Purpura (HSP), polyarteritis nodosa, Takayasu's arteritis, juvenile rheumatoid arthritis, systemic lupus erythematosus, inflammatory bowel disease, sarcoidosis, Sjogren syndrome, or Behcet disease should be considered [37, 66, 70–74].

### 5.7. Oncologic

Children with cancer are at increased risk for AIS as a result of their disease, subsequent treatment, and susceptibility to infection. Intracranial hemorrhage may complicate an intracranial tumor [2]. Leukemia and lymphoma create a hypercoagulable and hyperviscous state [75]. Treatment with L-asparaginase decreases antithrombin levels, and may trigger venous thrombosis in leukemic children concurrently receiving prednisone [73, 76]. Radiation therapy for optic chiasm gliomas or other sellar or suprasellar region tumours can cause vasculopathies that result in strokes which may be preceded by transient ischemic attacks (TIAs) beginning months to years after treatment [60, 77–79].

### 5.8. Trauma

Children who have experienced head and neck trauma are at risk of developing an ischemic event subsequent to dissection of the carotid or vertebral arteries. This can result from direct intraoral trauma delivered by a foreign object such as a pencil in the mouth or after tonsillectomy, and can also occur spontaneously [37, 66, 80–82]. Hyperextension or rotational injuries experienced during minor head trauma, motor vehicle collisions, sports such as wrestling, or even chiropractic manipulation can also result in strokes [60, 83, 84]. Symptoms of traumatic arterial dissection can be delayed by 24 hours, and the risk is greatest within a few days of the vascular injury [62, 83]. 

### 5.9. Drugs

Drug use, both illicit and prescribed, are a concern in the adolescent population. Cerebral infarcts and hemorrhage have been reported in patients abusing drugs such as amphetamines, ecstasy, cocaine, phencyclidine (PCP), and glue sniffing [85]. Stimulants and heroin can also cause vasculopathies predisposing to infarction [83]. Adolescent girls using oral contraceptives are at higher risk of cerebral venous thrombosis [86]. Overuse of ergot alkaloids in the treatment of acute migraines, are also associated with increased risk of ischemic events [87].

## 6. Management

The management of stroke in children is less-studied and largely extrapolated from the adult literature with the only randomized controlled trials for the treatment of acute stroke in children in the setting of SCD. However, generalizations and recommendations can still be made based on what is available and consensus statements. The emergency department management of stroke can be categorized into general supportive measures, diagnostic modalities, and treatment appropriate to the type of stroke identified.

Recommended universal supportive measures include the following: fever control, normalization of serum glucose, and maintenance of normal oxygenation as there is no evidence that supplemental oxygen is useful in nonhypoxic patients. Efforts should be made to ameliorate increased intracranial pressure (ICP), treat dehydration, and correct anemia. Control of systemic hypertension is recommended, but caution should be used as rapid reduction of blood pressure has been associated with worse neurological outcomes and larger infarcts in adults. Some experts do allow for mild permissive hypertension. There is no evidence to support prophylactic anticonvulsants without clinical or electroencephalography (EEG) evidence of seizures in children with AIS. However, anticonvulsants may be considered in children with hemorrhagic strokes and cerebral venous sinus thrombosis (CVST). Induced hypothermia is not recommended outside the context of a clinical trial [88].

### 6.1. Imaging and Testing

Noncontrast head computed tomography (CT) is sensitive for acute bleeding and should be obtained emergently to exclude a hemorrhagic cause of stroke. Despite increasingly advanced imaging techniques, the adult literature suggests that a lumbar puncture is still needed to rule out a subarachnoid hemorrhage (SAH) if one is not identified on CT and clinical suspicion remains high [89]. The sensitivity of CT in detecting SAH can be as low as 93%, and has been shown to decrease with time from onset of symptoms [90]. SAH can be present despite a normal neurological examination. If a hemorrhagic stroke is identified, magnetic resonance venography (MRV) should follow as 10% of hemorrhages in children are due to CVST [88]. If emergent magnetic resonance imaging (MRI) is available, it may also be used to exclude an acute intraparenchymal bleed or SAH. One series has suggested that MRI is as accurate as CT for the detection of hyperacute hemorrhage (i.e., <6 hours) [91].

Noncontrast CT is also the initial study for diagnosing AIS. Bland infarcts appear as low-density lesions within vascular territories, and CVST may present as linear densities in the deep and cortical veins. CT imaging after an AIS is usually normal within the first 12 hours after symptom onset, and MRI is a more sensitive test for early detection of an infarction [23, 88, 92]. Magnetic resonance arteriography (MRA) and MRV should also be carried out to confirm vessel patency and define the vascular anatomy. MRA will yield further information about blood flow, and MRV will more reliably identify CVST [23].

Catheter angiography (CA) yields the most precise detail of vascular anatomy of all imaging modalities, is superior to MRA and computed tomography angiography (CTA) for visualization of tertiary branches and small cerebral arteries, and can be performed in conjunction with endovascular therapy. It can identify signs of vasculitis or dissection, and medium or smaller artery abnormalities, which MRA may miss. However, CA is an invasive procedure that relatively few physicians have extensive experience performing in children. There is a relatively low likelihood of identifying a larger vessel abnormality with CA when MRA is negative [93]. However, CA should be strongly considered in cases with equivocal or negative findings on MR vascular imaging or where no other explanation for stroke is identified.

CT angiography (CTA) is another option for assessing vascular anatomy and relative cerebral blood flow. It may be used to identify arterial dissection causing AIS, and facilitates rapid assessment of vascular lesions requiring immediate surgery. Limitations of CTA include larger radiation doses than standard CT to facilitate the thin-slice profile necessary for high-quality CTA studies. The contrast required may also limit the volume of contrast that can be safely administered for subsequent, more definitive delineation by CA. MRA may be preferable to CTA, especially if the patient will subsequently be undergoing an MRI. However, CTA may still be useful in patients for whom MR is contraindicated. Other investigations to consider include ultrasound to evaluate the extracranial carotid circulation. Since cardiac anomalies are a significant risk factor for stroke in children, an ECG, chest radiograph, and transthoracic or transesophageal echocardiography may be useful.

There are no clearly established laboratory testing guidelines for the assessment of pediatric stroke. Laboratory assessment may include a variety of nonspecific blood tests and more specific laboratory tests looking for specific causes of stroke such as coagulopathies, hematological disorders, or vasculitides.  Table 2 provides a suggested list of laboratory and imaging tests to consider. One should also keep in mind that many thrombophilias are familial, and that other family members may also be affected and require evaluation. 

### 6.2. Treatment

Once the type of stroke is identified, treatment depends on the etiology. Hemorrhagic strokes may require medical management beyond supportive measures. Prevention of rebleeding includes correction of coagulation defects and hematologic disorders. Recombinant factor VIIa (rFVIIa) promotes hemostasis and has been shown to stabilize intracerebral hematomas and reduce hemorrhage volume. However, adult studies have not demonstrated improved survival or functional outcome at this time [94]. Further prospective studies in adults are still needed to determine if subsets of patients may benefit from this therapy, so it is likely too early to extrapolate this data to the pediatric population. 

Surgical management of hemorrhagic strokes is controversial. There may be benefit of early surgical evacuation in patients with clinical deterioration due to mass effect. Children may warrant more aggressive intervention given their lack of cerebral atrophy which, in older adults, could potentially accommodate some degree of hematoma expansion. Although a recent prospective, multicenter trial suggested that surgical evacuation of a supratentorial intraparenchymal hemorrhage does not improve chances of good recovery or moderate disability beyond best medical management, a recent meta-analysis does suggest that surgical evacuation is associated with a reduction in the odds of being dead or dependent [92, 95]. Other surgical options include stereotactic radiosurgery, microsurgical or endovascular techniques, and endoscopic surgical evacuation of the intracerebral hematoma or obliteration of aneurysms and AVMs [96–98]. Another surgical consideration is emergent splenectomy for intraparenchymal bleeding associated with idiopathic thrombocytopenic purpura [99].

Another goal specific to AIS management includes preventing a subsequent ischemic event. Medical options in the acute setting for prevention include anticoagulation with low molecular weight heparin (LMWH) or unfractionated heparin (UFH) (see Tables 3 and 4 for dosing). Although LMWH has reproducible pharmacokinetics and requires fewer monitoring tests, it cannot be reliably reversed with protamine, like UFH. One recent case series, however, suggests that rFVIIa may effectively reverse the effects of LMWH [100].

Although the practice of initiating short-term anticoagulation pending evaluation of stroke etiology in the adult population is no longer applied, recent guidelines have suggested that it may be prudent to start anticoagulation in children. This is because the likelihood of a child having an underlying condition that would benefit from anticoagulation (e.g., cervicocephalic arterial dissections, vasculopathy, unrecognized cardiac disease, and coagulopathy) is higher than in adults [88]. Anticoagulation is also often used in children with arterial dissection, dural sinus thrombosis, coagulation disorders, high risk of embolism, or progressive deterioration during the initial evaluation of a new cerebral infarction.

Long-term anticoagulation beyond the acute phase can be provided in the form of antiplatelet agents such as aspirin, clopidogrel, oral vitamin K antagonists like warfarin, or weekly subcutaneous LMWH injections. However, these measures can be initiated in consultation with the appropriate specialists after the initial management and stabilization are carried out in the emergency department setting.

Thrombolytic therapy in children with ischemic strokes must be carried out in a guarded and judicious manner. Published guidelines suggest that tPA may be considered in a select group of children with CVST, but could not make any further recommendations, including whether adult guidelines could be applied to adolescents who met adult eligibility criteria [88]. Although there are case reports and case series of IV recombinant tPA for children with strokes, there is little else upon which to base thrombolytic recommendations [101–104]. Despite anecdotal reports of successful endovascular thrombolysis and IV tPA use in children, there are other reports of high risks of hemorrhagic complication rates in children with systemic thrombolysis who receive IV tPA and inadequate evidence for deciding which patients are the best candidates [101–103, 105, 106]. An international multicenter study, “TIPS” (thrombolysis in pediatric stroke), is poised to begin with the goal of assessing the safety of IV tPA within 3 hours of AIS onset, and intra-arterial tPA within 3–6 hours of onset [107].

Management of stroke in children with sickle cell disease deserves special mention. Ischemic strokes should be treated with hydration and simple or partial exchange transfusion to achieve a hemoglobin SS fraction of less than 30% and a hemoglobin level not greater than 10 g per dL to avoid problems of hyperviscosity. Evaluation for a structural vascular lesion in children with sickle cell disease and a hemorrhagic stroke is reasonable. This is because there is often an underlying aneurysm with potential for rebleeding in adolescents with SCD who present with a SAH [108]. However, evaluation with CA to identify such aneurysms should be deferred until after reduction of the percentage of sickle hemoglobin because of concerns that CA might facilitate sickling [88]. Surgical revascularization procedures may be considered as a last resort in children with sickle cell disease who have persisting cerebrovascular dysfunction despite optimal medical management [88].

Rapid transfer to a tertiary pediatric center is indicated. In situations where further information or guidance is desired, a call to a pediatric stroke telephone consultation service like 1-800-NOCLOTS may be useful. This is a free service to physicians seeking advice on management of children with stroke based on the “best available evidence.” The service was established in 1994, and is staffed by pediatric hematologists and neurologists based at the Hospital for Sick Children in Toronto, Canada. Calling this service is not only a means of obtaining assistance, but helps with the collection of information for future study [109].

## 7. Conclusions

Strokes in children are being recognized more frequently as diagnostic aids develop and clinician recognition improves. However, because the incidence is still low relative to adult strokes, and children are distinctly different from adults, it remains a challenge to create evidence based diagnostic and treatment guidelines. Due to the low incidence of this disease, future stroke research needs to be pursued with a collaborative effort both nationally and internationally. RCTs specific to children are clearly needed to better establish the safety and efficacy of both acute and preventative treatments. The long-awaited and highly anticipated TIPS trial will lead the way with other studies to improve care for children [107]. Until then, stroke should remain a strong consideration in children with concerning signs and symptoms and significant risk factors, and the best available evidence should be utilized in providing optimal medical care.

## Figures and Tables

**Table 1:** Clinical presentation of pediatric ischemic and hemorrhagic strokes.

	Ischemic		Hemorrhagic	
	Earley et al. 1998 [1]	DeVeber et al. 2000 [18]	Earley et al. 1998 [1]	Meyer-Heim and Boltshauser et al. 2003 [26]

Hemiparesis or focal CNS deficit	94%	51%	21%	16%
Change in mental status	28%		88%	52%
Headache	22%		59%	76%
Seizure	16%	48%	29%	28%
Speech disorder, incl. aphasia		17%		8%
Vomiting				48%
Nausea				20%
Somnolence				12%
Visual impairment				12%
Neck pain				8%
Fever/prodrome	35–40%		35–40%	

**Table 2:** Laboratory and diagnostic testing considerations for the acute pediatric stroke patient.

Additional laboratory tests to consider	Additional tests to consider
Liver function	Brain MRI
ESR	MRA
CRP	(i) Intracranial vessels
Pregnancy	(ii) Extracranial great vessels (neck)
ANA	MRV
Lupus anticoagulant	Diffusion weighted imaging (DWI)
Anticardiolipin antibody	CT angiogram
Beta-2 glycoprotein-1 antibody	(i) Intracranial vessels
Activated protein C resistance	(ii) Extracranial great vessels (neck)
Factor V Leiden mutation	Contrast transthoracic echo (TTE)
Protein S/C function	Cerebral angiogram
Antithrombin III	Contrast transesophageal echo (TEE)
Prothrombin gene mutation	Electroencephalogram (EEG)
Homocysteine level	Lumbar puncture
Methyltetrahydrofolate reductase allele (MTHFR)	Holter monitoring
Fibrinogen disorder	Transcranial doppler
Plasminogen activator inhibitor disorder	
Factor VII/VIII elevation	
Factor XII deficiency	
Plasma amino acids/urine amino and organic acids	
Serum and CSF lactate/pyruvate	
Hemoglobin electrophoresis	
Triglycerides/cholesterol	
Lipoprotein (a)	
Miscellaneous bacterial, fungal, spirochetal, parasitic,	
viral, and rickettsial tests (i.e., Lyme, PPD, VDRL)	
Serum and CSF varicella titers	
HIV titers	

Adapted from Younkin [23] and Deveber [92].

**Table 3:** Protocol for using LMWH in children.

Preparation	Initial treatment dose	Initial prophylactic dose
Reviparin, body weight-dependent dose, units/kg per 12 h		
<5 kg	150	50
>5 kg	100	30

Enoxaparin, age-dependent dose, mg/kg per 12 h		
<2 months old	1.5	0.75
>2 months old	1.0	0.5
Dalteparin, all-age pediatric dose, units/kg per 24 h	129 ± 43	95 ± 52

Tinzaparin, age-dependent dose, units/kg		
0 to 2 months old		275
2 to 12 months old		250
1 to 5 years old		240
5 to 10 years old		200
10 to 16 years old		275

Adapted from Roach et al. [88].

**Table 4:** Protocol for systemic heparin administration and adjustment in children.

Stage	aPTT (sec)	Dose (units/kg)	Hold (min)	Rate change (%)	Repeat aPTT
(I) Loading dose		75 IV over 10 min			
(II) Initial maintenance dose					
Infants < 1 yo		28/h			
Children > 1 yo		20/h			
(III) Adjustment	<50	50	0	10	4 h
	50–59	0	0	10	4 h
	60–85	0	0	0	Next day
	86–95	0	0	−10	4 h
	96–120	0	30	−10	4 h
	>120	0	60	−15	4 h
(IV) Obtain blood for aPTT 4 h after heparin load and 4 h after every infusion rate change					
(V) When apt values are in therapeutic range, perform daily CBC and apt measurement					

Adapted from Roach et al. [88]
